# Shape-Selective Supramolecular Capsules for Actinide Precipitation and Separation

**DOI:** 10.1021/jacsau.3c00793

**Published:** 2024-02-12

**Authors:** Joseph O’Connell-Danes, Bryne T. Ngwenya, Carole A. Morrison, Gary S. Nichol, Lætitia H. Delmau, Jason B. Love

**Affiliations:** † EaStCHEM School of Chemistry, University of Edinburgh, Edinburgh EH9 3FJ, U.K.; ‡ School of Geosciences, University of Edinburgh, Edinburgh EH9 3FE, U.K.; § Radioisotope Science and Technology Division, Oak Ridge National Laboratory, Oak Ridge, Tennessee 37831, United States

**Keywords:** f-elements, anion recognition, radiochemistry, nuclear chemistry, supramolecular

## Abstract

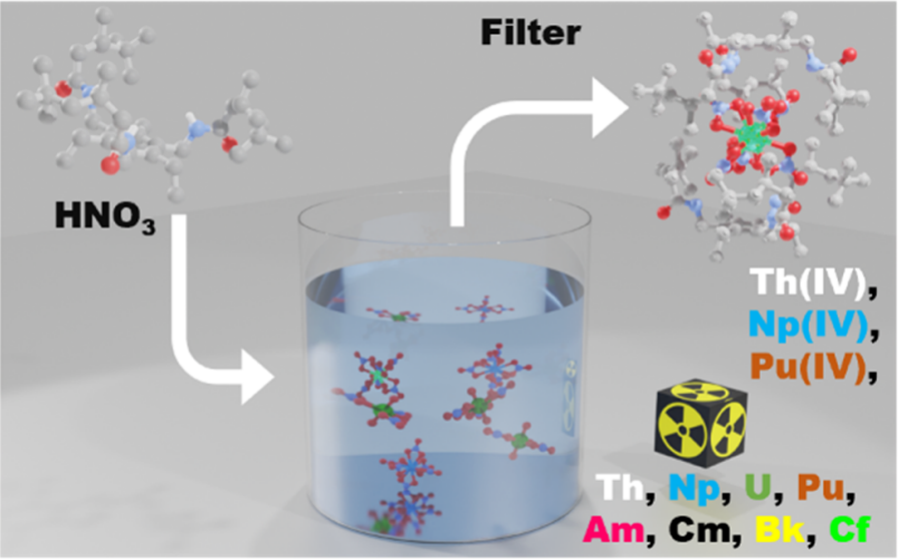

Improving actinide separations is key to reducing barriers to medical and industrial actinide isotope production and to addressing the challenges associated with the reprocessing of spent nuclear fuel. Here, we report the first example of a supramolecular anion recognition process that can achieve this goal. We have designed a preorganized triamidoarene receptor that induces quantitative precipitation of the early actinides Th(IV), Np(IV), and Pu(IV) from industrially relevant conditions through the formation of self-assembled hydrogen-bonded capsules. Selectivity over the later An(III) elements is shown through modulation of the nitric acid concentration, and no precipitation of actinyl or transition-metal ions occurs. The Np, Pu, and Am precipitates were characterized structurally by single-crystal X-ray diffraction and reveal shape specificity of the internal hydrogen-bonding array for the encapsulated hexanitratometalates. This work complements ion-exchange resins for 5f-element separations and illustrates the significant potential of supramolecular separation methods that target anionic actinide species.

## 1 Introduction

Increasing demand for actinide elements in medicine, industry, and space exploration, alongside renewed interest in nuclear power and associated spent fuel reprocessing and partitioning has highlighted the need for improved separation technologies.^1−5^

Separation methods that target anionic metal compounds (metalates) are some of the most selective found for transition metals.^6−8^ In this context, the exploitation of highly controllable, noncovalent interactions to form supramolecular assemblies provides unparalleled specificity for anion recognition.^9−12^ These structures allow for extraction from highly acidic media and permit facile release of the separated metal anion, both of which pose significant issues in more conventional separation processes that exploit the formation of inner-sphere metal–ligand bonds.^13^ Despite these advantages, few examples of supramolecular anion separations for f-elements exist, and, to our knowledge, there are no examples for the actinide elements.^14−18^

Current industrialized separations for actinide elements rely on solid sorbent materials and solvent extraction techniques.^19−21^ Anion-exchange resins functionalized with quaternary ammonium or sulfonic acid functional groups can selectively separate actinides, whereas chromatographic resins containing the diglycolamide ligand TODGA have demonstrated impressive separations for a range of actinide and lanthanide elements.^22−26^ While the formation of actinide metalates is implicated in these processes, the precise mechanisms are not wholly understood. Impregnated polymer resins containing acidic alkylphosphorus extractants have also been developed that show significant selectivity for actinide cations generated in the production of ^252^Cf and ^238^Pu, among other isotopes.^23,27^ However, challenges exist regarding resin stability, scalability, and disposal of the resulting contaminated waste material.

Solvent extraction processes for actinide cation separations have been designed around a range of extractants with various chemical functionalities.^28−30^ Processes such as TALSPEAK use a combination of acidic extractants (e.g., phosphoric acids, polyaminocarboxylates, and hydroxypyridones), and exploit the difference in covalency of the 4f and 5f elements to achieve selectivity.^31−33^ Although cation-targeting solvent extraction systems are capable of very high selectivity between 5f and non-5f elements, the small differences in metal–ligand bonding result in limited selectivity within the 5f period. Bis-triazine extractants such as BTBP/BTP have achieved some of the highest single-step separation factors between Am(III) and Eu(III),^34^ but difficulties in stripping the extracted metal combined with poor kinetics have limited their application.^35^ Diglycolamide extractants such as TODGA and TEHDGA display high affinity for Ln/An(III) metal ions over An(VI) metal ions, and excellent extraction kinetics and distribution ratios are evident.^36^

Recently, we reported a preorganized triamidoarene platform which, under acidic, biphasic conditions, acts as a host to selectively precipitate the hexanitratometalates of the early rare-earth elements through the formation of intra- and intermolecular hydrogen-bonded capsules.^17^ Driven by the differences in the stability of the encapsulated hexanitratometalates, this system demonstrated some of the highest separation factors between the early and late rare-earth elements. In this work, we apply this supramolecular strategy to present the first examples of molecular metalate separation for the actinide series.

## 2 Results and Discussion

### 2.1 An(IV) Capsule Precipitation and Characterization

**Figure 1:**
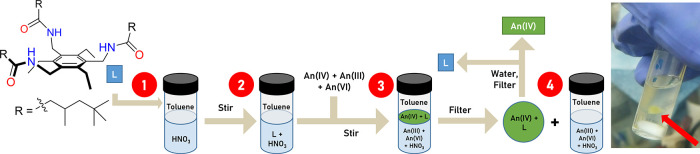
(Left) Schematic of the precipitation of An(IV) nitratometalates by **L** from biphasic nitric acid/toluene mixtures and the release of the loaded metal using water. (Right) ^238^Pu-containing precipitate on an organic/aqueous interface.

The tripodal amido-arene **L** (Figure 1), synthesized according to our previous report,^17^ was dissolved into the aqueous phase of a biphasic mixture of toluene and 8 M HNO_3_ (Figure 1, steps 1 and 2). Upon addition of an aqueous solution of An(IV) (An = Th, ^237^Np, and ^238/239^Pu) in nitric acid and agitating the vial, a solid precipitate is formed immediately at the interface between the organic and aqueous phases (Figure 1, step 3) and, in the case of Np and Pu, complete discoloration of the aqueous phase is observed (Figure 1, right). Analysis of the aqueous phases by inductively coupled plasma-mass-spectrometry (ICP-MS) (Th) or α/α spectroscopy (Np, Pu) confirms that near-complete precipitation of the metals occurs.

**Figure 2:**
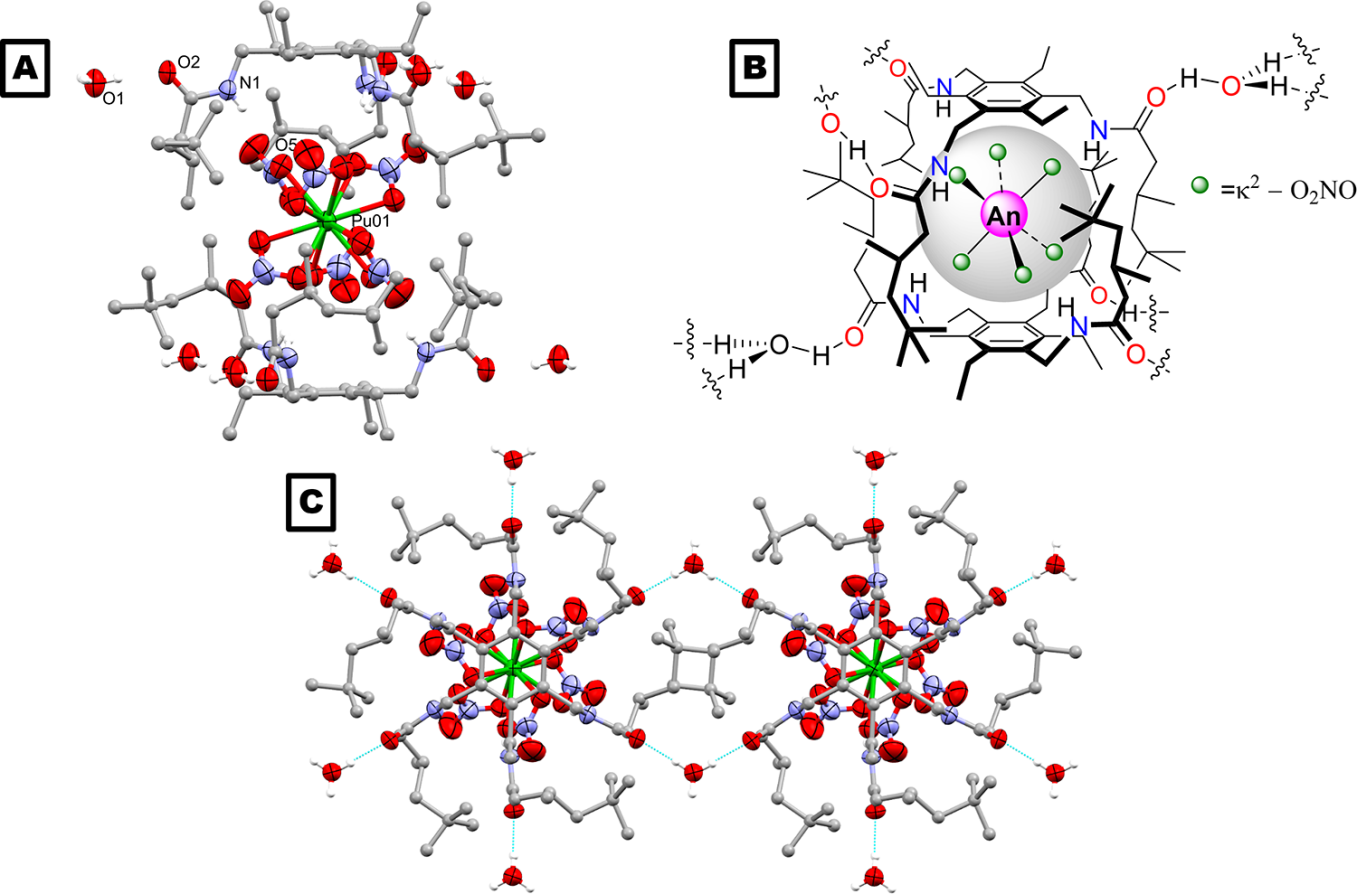
(A) Capsule geometry obtained from the X-ray crystal structure of **1-Pu** (side-on view). For clarity, all hydrogen atoms except those involved in hydrogen bonding and a disorder component of the amide arm are omitted (where shown, thermal displacement ellipsoids are drawn at 50% probability). N–H and O–H hydrogen atoms were located in the difference Fourier map and (O1) is 1/3% occupied on a crystallographic special position. Atom colors: Pu = green; oxygen = red; nitrogen = blue; carbon = silver; hydrogen = white. (B) ChemDraw representation of the capsular hexanitratometalate complex [{An(IV)(κ^2^-NO_3_)_6_}⊂(L_2_)(H_3_O)_2_]_n_ (C) Top-down view of a two-capsule segment of the extended structure highlighting the bridging H_3_O^+^ units.

The precipitates **1-Th**, **1-Np**, and **1-Pu** were isolated by filtration (Figure 1, step 4) and dissolved in hot acetonitrile, from which diffraction-quality crystals formed through slow-cooling. Note the X-ray crystal structure for **1-Pu** was grown from a solution of ^239^Pu, as the crystals grown in solutions of ^238^Pu tended to crack due to the high heat generated by this isotope. All other precipitation experiments detailed below were carried out by using ^238^Pu. The X-ray crystal structures of the precipitates formed for all three metals are isostructural, displaying hexanitratometalate anions An(NO_3_)_6_^2–^ encapsulated by two amido-arene receptors to form a supramolecular capsule (Figure 2, **1-Pu**, see also the Supporting Information, Figures S2 and S3). The resulting negative charge of the metalate is balanced by two H_3_O^+^ ions which provide bridging links to neighboring capsules. This packing motif extends to form two-dimensional (2-D) sheets. The formation of this extended structure provides an explanation for the observed precipitation, with the solid being insoluble in both the organic and aqueous phases. The internal capsule structure is similar to that seen previously in the encapsulation of the Ln(NO_3_)_6_^3–^ anions, showing an *ababab* configuration commonly observed in statically geared hexa-alkylarene platforms.^17,37,38^

Comparing the geometries across the group (Th to Pu; Table 1) shows that the κ^2^-nitrate is bound asymmetrically in all instances. A considerable shortening of the metal–oxygen bond distances from 2.573(3) and 2.545(3) Å in **1-Th** to 2.508(3) and 2.471(2) Å in **1-Pu** is seen, in line with those reported previously.^39^ Comparing **1-Th** with its 4f group congener, **1-Ce** (Supporting Information, Figure S1), shows a shortening of the M–O bond lengths, as expected for the increased cationic charge on Th^4+^ compared with Ce^3+^. Surprisingly, the intracapsule amide-N to nitrate-O N(H)···ONO_2_ hydrogen bond distances are considerably longer in **1-Th** than in **1-Ce**. Quantum theory of atoms in molecules (QTAIM)^40^ analysis of the electron density of the DFT-optimized structures indicates that the intracapsular hydrogen-bonding interaction in **1-Th** is 2.7 times weaker than in **1-Ce** (Supporting Information, Table S7). The strength of the intracapsule hydrogen-bonding interaction decreases moving across the period from Th to Pu, with the same comparison of the electron density in the bond critical points indicating that the N(H)···ONO_2_ bond in **1-Pu** is 1.4 times weaker than that in **1-Th**. This reduction in bond strength results in the capsules becoming more prolate, as measured by the centroid ring···ring distance (Table 1). The decrease in the magnitude of intracapsule hydrogen bonding can be rationalized through the relative changes in the electron density at the bond critical points for the M–O bonds, which increase going from the 4f and 5f metals and across the 5f series. Qualitative analysis of the electron density difference plots (Supporting Information, Figure S5) shows an increase in bonding electron density in the M–O bonds, and a decrease in the associated hydrogen bonds between **1-Ce** and **1-Th**, and **1-Th** and **1-Pu**. Both analyses suggest that more of the electron density from the nitrate oxygen atom is involved in bonding with the An(IV) metal than with the hydrogen bond to the capsule as the element changes down a group (4f to 5f) and across the row of the periodic table.

**Table 1:** Bond Lengths (Å) from the X-ray Crystal Structures of **1-Th**, **1-Np**, **1-Pu**, and **1-Ce**

distance (Å)	Ce	Th	Np	Pu
M–O	2.6409(19)	2.573(3)	2.520(2)	2.508(3)
M–O	2.6157(19)	2.545(3)	2.483(2)	2.471(2)
N(H)···ONO_2_	2.823(4)	3.249(4)	3.333(3)	3.348(4)
O(H)···O	2.435(4)	2.562(3)	2.5849(14)	2.588(4)
Centroid (ring···ring)	10.818(2)	11.875(2)	11.9867(17)	11.9973(4)

The Raman spectra of the bulk precipitates of **1-Th**, **1-Np**, and **1-Pu** (Supporting Information, Figure S6) show the symmetric stretching vibrations (ν_s_) associated with the metal-bound NO_3_^–^ anions at 1032, 1034, and 1037 cm^–1^ respectively, in line with literature values.^39^ The IR spectrum of **1-Th** (Supporting Information, Figure S7) shows a shift in the C=O stretch from 1638 cm^–1^ in **L** to 1527 cm^–1^ in **1-Th**, which is indicative of the interaction of the carbonyl oxygen with the hydronium ion and is similar to the shift seen for the related lanthanum capsule [La{NO_3_}_6_⊂(H_3_**L**_2_)].^17^ The IR spectrum also displays an additional absorption band at 3340 cm^–1^ that is attributable to the bridging hydronium ion.^41^ The vibrational spectra indicate that the precipitate and single crystals are structurally coherent.

### 2.2 Selective An(IV/VI) Precipitation

**Figure 3:**
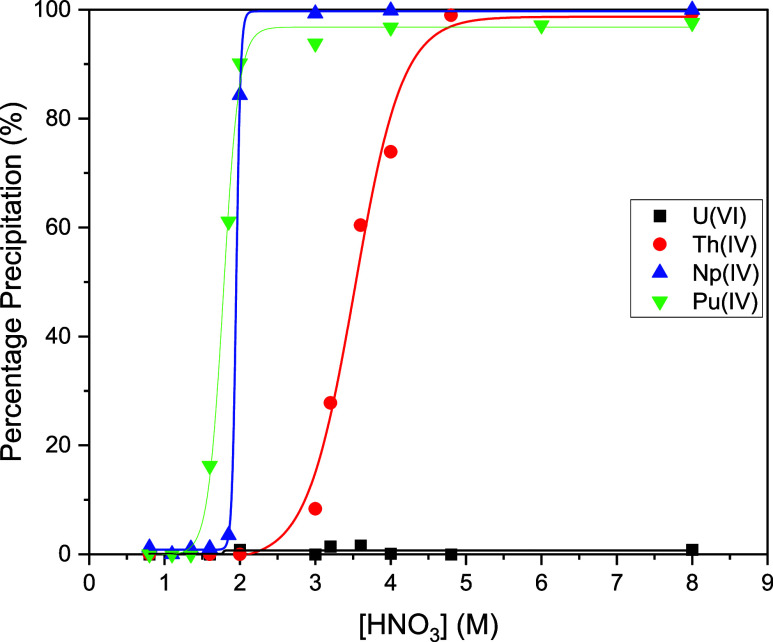
Precipitation arising from single-metal solutions of ^237^Np (474 ppm) and ^238^Pu (486 ppm) and mixed-metal solutions of U and Th (595 and 580 ppm, respectively) in 0.8–8.0 M HNO_3_/toluene equal-volume biphasic mixtures after the addition of **L** (10-fold excess **L** relative to metal) at 298 K. Lines have been drawn for ease of comprehension.

Precipitation experiments conducted for mixed-metal solutions of Th(IV) and U(VI) and single-metal solutions of Np(IV) and Pu(IV) with excess **L** in a toluene/nitric acid biphasic mixture, conducted over 0.8–8.0 M HNO_3_, highlights a dependency on nitric acid concentration (Figure 3). Precipitations of **1-Th**, **1-Np**, and **1-Pu** follow a sigmoidal trend which is observed extensively in metalate, “anion-swing”-type processes for the extraction of transition metals. This is the first report of this process in the extraction and precipitation of actinides.^42^

The dependency on the nitric acid concentration for precipitation differs between the elements. Sharp increases in precipitation are observed above 1.6 M HNO_3_ for Pu and 1.9 M HNO_3_ for Np, with quantitative Np and 97% Pu precipitation observed at concentrations above 2.0 M. In contrast, significant Th precipitation is only seen at concentrations above 3 M HNO_3_, with 98% precipitation seen above 4.8 M HNO_3_. No U(VI) precipitation occurred at any acid concentration. This nitric acid dependency on the selectivity of precipitation follows trends exhibited by commercialized chromatographic resin processes.^20^

From the combination of the precipitation data and structural information obtained, it is apparent that the formation of the hexanitratometalate anion is the key requirement for its encapsulation and subsequent precipitation. The rigidly preorganized and highly symmetric nature of the tripodal amide, particularly with respect to the positioning of the N–H hydrogen bond donors within the interior of the capsule, results in shape specificity for the highly symmetric, 12-coordinate metalates of Th(IV), Np(IV), and Pu(IV).

In contrast, in acidic matrices, uranium forms U(VI) uranyl complexes, which are highly stable even in the presence of mild reducing agents like HNO_2_. The axial oxygen atoms of these ions result in a noncomplementary anion shape for the internal structure of the capsule and, as a result, no uranium(VI) precipitation by **L** is observed, even at very high nitrate concentrations. Neptunium speciation in acids is more complex, with Np(VI)O_2_^2+^, Np(V)O_2_^+^, and Np(IV) coexisting in equilibrium. In general, the equilibrium is dominated by Np(VI) and Np(V) actinyls,^43^ with their relative concentrations in 8 M HNO_3_ being 71% Np(VI), 10% Np(V), and 19% Np(IV) by UV–vis/NIR spectroscopy (Supporting Information, Figure S8). The addition of **L** to this mixed oxidation state solution results in 26% precipitation of **1-Np** (Supporting Information, Figure S11), which aligns with the total amount of Np(IV) in solution, assuming that the small amount of Np(V) disproportionates to Np(IV) and Np(VI). The addition of hydrogen peroxide to this solution generates the reducing agent HNO_2_, which drives the complete conversion of both Np(VI) and Np(V) to Np(IV); the resulting [Np(IV)(NO_3_)_6_]^2–^ metalate anion is precipitated quantitatively by **L.** This experiment highlights how further selectivity in separation can be achieved through careful control of the redox chemistry of the actinide solutions.

The metals can be readily and quantitatively stripped from the precipitated solids. Isolating the precipitate and washing it with HNO_3_ removes any nonprecipitated metal. Subsequent washing with water interrupts the supramolecular hydrogen-bonding interactions in the capsules and liberates the metalate anions into solution (Supporting Information, Figure S11). The precipitated ligand can subsequently be recovered by filtration for further use.

### 2.3 An(III) Precipitation

**Figure 4:**
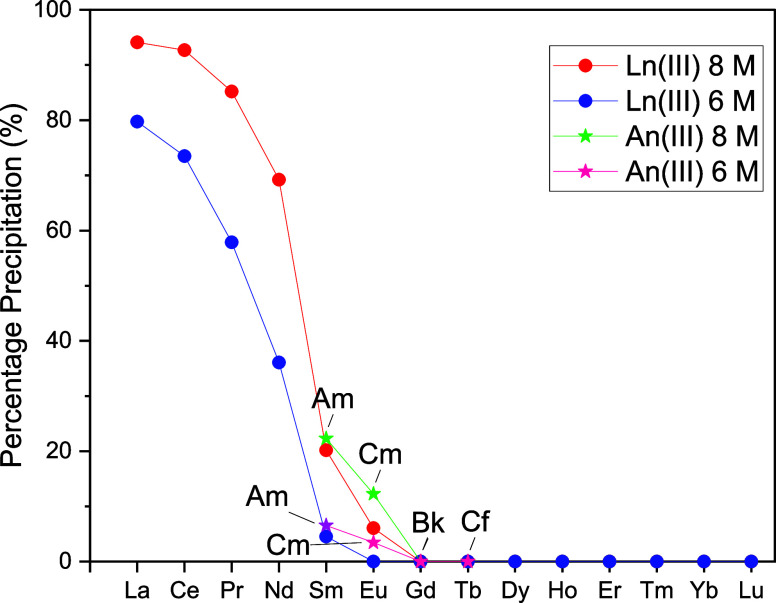
Precipitation arising from a mixed rare-earth solution (∼500 ppm), single-metal solutions of ^243^Am and ^245/248^Cm (230 and 265 ppm respectively), and mixed-metal solutions of Bk/Cf (373 and 245 ppm, respectively) in 6.0 to 8.0 M HNO_3_/toluene equal-volume biphasic mixture after the addition of **L** (5-fold excess L relative to metal) at 298 K. Rare-earth precipitations are plotted using data that were previously used to generate a figure found in ref (17).

The precipitation behavior of Am(III), Cm(III), Bk(III), and Cf(III) in 8.0 and 6.0 M HNO_3_ follows the same trend as the data reported previously for the lanthanide series,^17^ but with the percentage precipitation values aligning with the corresponding left-diagonal element (i.e., Am(III) aligns with Sm(III), see Figure 4). The limited precipitation of Am(III), at 22.3% in 8 M HNO_3_, compared with An(IV) reflects the dramatically lower stability of the hexanitratometalates of the An(III) elements compared with that of the An(IV) elements, which arises as a result of the weaker metal-nitrate bonding in aqueous solution. Based on the previous investigations into lanthanide precipitation by **L**, little or no precipitation of any of the trans-plutonium elements would be expected at acid concentrations below 6 M HNO_3_.^17^ Thus, the decrease in precipitation seen from Am to Cf likely reflects the decreasing propensity to form 12-coordinate nitratometalate complexes due to the contraction in the ionic radius upon traversing the An series.

### 2.4 X-ray Structure of Am(III) Precipitate **1-Am**

**Figure 5:**
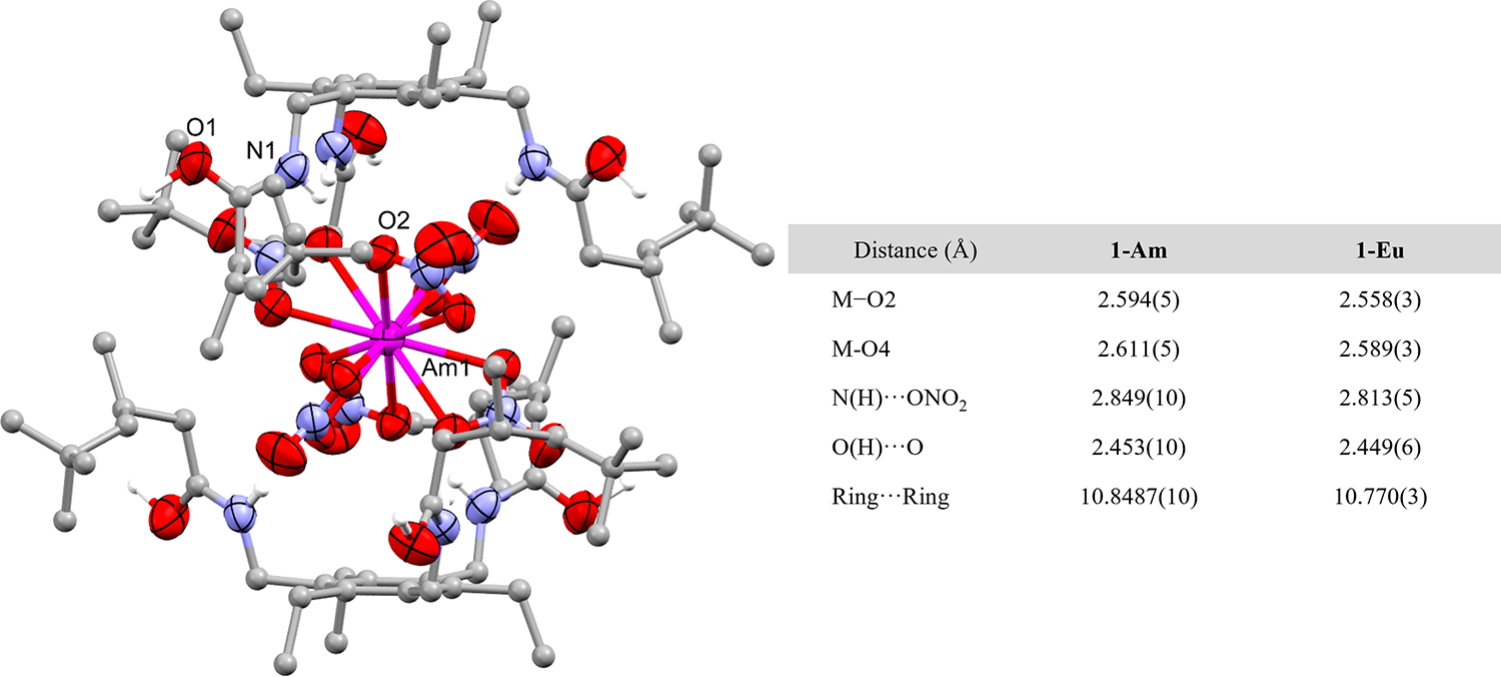
(Left) X-ray crystal structure of **1-Am** (side-on view). For clarity, all hydrogen atoms except those involved in hydrogen bonding and a disorder component of the amide arm are omitted (where shown, thermal displacement ellipsoids are drawn at 50% probability). N–H and O–H hydrogen atoms were located in the difference Fourier map, and (O1)H is 1/2% occupied on a crystallographic special position. Atom colors: Am = pink; oxygen = red; nitrogen = blue; carbon = gray; hydrogen = white. (Right) Selected bond lengths from the X-ray crystal structures of **1-Am** and **1-Eu**.

The precipitate of **1-Am** was isolated by filtration and diffraction-quality crystals were obtained through slow evaporation of a solution of **1-Am** in acetonitrile. The X-ray crystal structure of **1-Am** (Figure 5) is isostructural with the structures of the Ln(III) complexes, **1-Ce** and **1-Eu** (Supporting Information, Figures S1 and S4). Comparison of the key bond lengths in the structures of **1-Am** and 4f group congener **1-Eu** provides insight into the observed precipitation trends between the Ln(III) and An(III) metals. The metal–oxygen bonds in **1-Am** are slightly longer than in **1-Eu**, which suggests a larger, less sterically crowded metalate that would form more easily in nitric acid, and thus be more likely to be precipitated on interaction with the receptor **L**. This is reflected in the degree of precipitation of Am, which more closely aligns with the diagonally related Sm rather than the group congener Eu. This trend runs contrary to that observed in the An(IV) series, which we speculate reflects the complicated interplay between steric and electrostatic considerations in the stability of the metalate.

### 2.5 Selectivity over Fission and Corrosion Products

In addition to the wide array of f-elements in solution, separation processes for the production of high-purity actinide radioisotopes also require a high degree of selectivity over a myriad of fission products (e.g., ruthenium, zirconium, and niobium) and corrosion products (e.g., iron, nickel, aluminum). The precipitation selectivity of the amide over these metals was further investigated through the addition of **L** to biphasic 8 M HNO_3_/toluene solutions containing a diverse range of metals, including those identified as fission and corrosion products. Within error, no precipitation of any of the metals investigated is observed (Supporting Information, Figure S13). This selectivity again highlights the high specificity of the capsules for 12-coordinate hexanitratometalates. Outside of the f-block, there are currently only three reported 12-coordinate hexanitratometalates, one for each of barium, lead, and strontium, of which none are formed in acidic solutions.^44,45^

## 3 Conclusions

A shape-selective supramolecular receptor has been shown to be highly effective and selective for actinide separation under industrially relevant conditions. The system exhibits selectivity based on the metal oxidation state, size, and maximum accessible coordination number, as a result of the absolute specificity for a single anionic species in the form of the 12-coordinate hexanitratometalate. This allows for some separation of Th(IV) and complete separation of the other An(IV) metals, An(IV) metals from An/Ln(III) metals, and complete selectivity for the f-elements over any transition or base metal. These results, coupled with the structural insights into the assemblies, highlight the powerful potential for targeting the underexplored metalates in f-element separations chemistry. This method could have significant potential for other societally important separations, such as in the recycling of permanent magnets in end-of-life electronics. More broadly, we envision the development of further anion-specific receptors which could allow for the targeting of alternative f-element species such as hexathiocyanato, hexachlorido, or pentanitrato complexes, allowing for access to entirely new selectivity regimes.^46,47^

## 4 Experimental Section

All aqueous solutions were prepared with deionized water obtained from a Milli-Q Gradient A10 system (18 MΩ). Nitric acid (68%) was ACS grade and was received from Fisher Scientific. The tripodal amido-arene **L** was prepared according to previously reported synthesis.^17^ The uranyl nitrate and thorium nitrate salts were obtained from Strem Chemicals. The neptunium-237 and plutonium-238 nitrate solutions were obtained by consolidating and purifying small sample solutions obtained during the course of monitoring processes pertaining to the Pu-238 Supply Program. The berkelium-249 and californium-249 nitrate solutions were also consolidations of side-stream solutions from the Cf-252 production program, which were subsequently purified. Plutonium-239, americium, and curium solutions were obtained in-house. All stock solutions were analyzed prior to use in this study.

### 4.1 Equipment and Instrumentation

#### 4.1.1 Facilities

With the exception of the uranium and thorium work, all of the work reported here was performed at the Radiochemical Engineering Development Center (REDC) in negative-pressure glove boxes.

#### 4.1.2 Metal Content Analysis

Multiple radiometric methods were used in these studies. The gross α counting was performed with a Gross-Alpha Protean MPC 2000 Gas Flow Proportional Counter. The α spectrometry was performed with a Canberra Model 7401 Alpha Spectrometer, and the γ spectrometry was performed with high-purity Ge detectors (ORTech and Canberra). α and γ spectra were processed by using Canberra Genie2K Acquisition and Analysis Software. Interferences between γ lines are tabulated in a reference table integrated in the software and are taken into account when the spectra are processed. The liquid scintillation counting (LSC) was performed with a Tri-Carb 4910TR liquid scintillation counter (PerkinElmer); samples were prepared using 5 mL of Ultima Gold liquid scintillation cocktail in standard polyethylene scintillation vials (20 mL). All analytical results were provided by the Nuclear Analytical Chemistry and Isotopes Laboratory. Uncertainties associated with these results are ±10%. ICP-MS analysis was carried out on an Agilent 7800 Single Quadrupole Inductively Coupled Plasma Mass Spectrometer. Samples in 2% nitric acid were taken up by a peristaltic pump at a rate of 0.3 rps into a MicroMist nebulizer and a quartz Scott-type spray chamber. Argon plasma conditions were 1550 W RF power and gas flows of 15, 1.07, and 0.9 L min^–1^ for plasma, auxiliary, and nebulizer flow, respectively.

#### 4.1.3 IR and Raman Spectroscopy

Raman spectra were collected by using a Renishaw inVia micro-Raman spectrometer with an excitation wavelength of 785 nm (500 mW). Point measurements were collected with a 50× objective and a Leica microscope. Spectra were acquired from 200–1800 cm^–1^ with a 1200 L/mm diffraction grating and spectral resolution of ∼3 cm^–1^. The laser power was set at 5% and each spectrum is the sum of 10 accumulations and a 5 s exposure time. Samples were placed in a glass slide cavity and sealed with a 1 mm thick quartz top slide. Fourier transform infrared (ATR FT-IR) measurements were performed on a PerkinElmer 65 FT-IR spectrometer over the range 4000–500 cm^–1^.

#### 4.1.4 Absorption Spectroscopy

A QEPro spectrophotometer (Ocean Insight, Orlando, FL) was used for ultraviolet (UV)–vis–NIR absorption measurements. Each vis–NIR spectrum resulted from an average of five scans, including absorbance measurements every 0.78 nm (325–1115 nm). The vis–NIR measurements had an optical resolution of ∼2 nm with a 25 μm slit. NIR spectra were measured with an Ocean Insight NIRQuest instrument at 1.5 nm increments from 900 to 1690 nm. Quantification of Np and Pu oxidation states was calculated from previously reported molar absorptivities.^48,49^ The quantification of Np(IV) in the nonstabilized solution was done indirectly by subtraction of the Np(VI) and Np(V) concentrations from the total concentration (as determined by α–α spectrometry) as the Np(IV) bands could not easily be deconvoluted from the other species present. Np(VI) was quantified based on the 1230 nm band and a molar absorptivity of ε = 19 M^–1^ cm^–1^, Np(V) was quantified based on the 980 nm band and a molar absorptivity of ε = 290 M^–1^ cm^–1^.

#### 4.1.5 NCI Plots and QTAIM Analysis

Geometry optimization (atom-only) calculations were performed using CASTEP v17.21^50^ for **1-Ce**, **1-Th**, **1-Np**, and **1-Pu** following editing to remove the positional disorder from the affected atomic positions. The basis set was constructed from on-the-fly pseudopotentials and plane waves expressed to 650 eV, combined with the generalized gradient approximation PBE.^51^ Brillouin zone sampling was below 0.05 Å^–1^. Geometry optimization criteria: energy tolerance = 2 × 10^–5^ eV atom^–1^, max force = 0.05 eV Å^–1^, max atomic displacement = 2 × 10^–3^ Å. Charge density cubes were then generated using the CASTEP2CUBE facility and processed using the CRITIC2 code^52,53^ for QTAIM analysis.^54^

#### 4.1.6 X-ray Crystallography

##### 4.1.6.1 **1-Th**

Diffraction-quality crystals were grown from a supersaturated solution of **1-Th** in acetonitrile. The solution was heated to 60 °C for 0.5 h and then allowed to cool slowly and left to stand for several days to give the crystals as colorless blocks.

##### 4.1.6.2 **1-Np**

Diffraction-quality crystals were grown from a supersaturated solution of **1-Np** in acetonitrile. The solution was heated to 60 °C for 0.5 h and then allowed to cool slowly and left to stand for several days to give the crystals as colorless blocks.

##### 4.1.6.3 **1-**^**239**^**Pu**

Diffraction-quality crystals were grown from supersaturated solution **1-**^**239**^**Pu** in acetonitrile. The solution was heated to 60 °C for 0.5 h and then allowed to cool slowly and left to stand for several days to give the crystals as brownish blocks.

##### 4.1.6.4 **1-Ce**

Colorless blocks were grown by slow evaporation of a supersaturated solution (10 mM) of **1-Ce** in acetonitrile over a period of several months.

##### 4.1.6.5 **1-Am**

Colorless blocks were grown by slow evaporation of a supersaturated solution of **1-Am** in acetonitrile over a period of several days.

##### 4.1.6.6 **1-Eu**

Colorless blocks were grown by slow evaporation of a supersaturated solution (10 mM) of **1-Eu** in acetonitrile over a period of several months.

##### 4.1.6.7 **1-Th**, **1-Ce**, and **1-Eu**

X-ray data were collected at 120 K on a Rigaku Oxford Diffraction Supernova diffractometer by using Cu Kα radiation (λ = 1.5418 Å). The structure was solved by direct methods using ShelXT and refined using a full-matrix least-squares refinement using ShelXL, both within the Olex2 (v1.5) software. Crystallographic data are presented in Supporting Information, Tables S3 and S4.

##### 4.1.6.8 **1-Np**, **1-**^**239**^**Pu**, **1-Am**

X-ray data were collected with the Cryostream switched off, and X-ray diffraction measurements were performed on a Bruker D8 Venture diffractometer equipped with an Ims 3.0 MoKα X-ray source (λ = 0.71073 Å). Apex IV software was used for data collection and unit cell determination. Both crystals were mounted to a Mitegen Cryoloop with quick-setting epoxy. After the epoxy had set, a plastic sheath was epoxied onto the mitogen Cryoloop for further contamination control. This sheath precluded the use of a Cryostream for low-temperature data collection. The sheath also precluded the capture of high-quality crystal images, preventing the accurate determination of crystal sizes. The structure was solved by direct methods using ShelXT and refined using a full-matrix least-squares refinement using ShelXL, both within the Olex2 (v1.5) software. Crystallographic data are presented in Supporting Information, Tables S1, S2, and S5.

### 4.2 Variable Acid Precipitation Experiments

#### 4.2.1 Precipitation Procedure for Th and U

Nitric acid solutions (0.8–8 M) were prepared by dilution of concentrated nitric acid with ultrapure deionized water. Mixed-U/Th solutions (0.0025 M) were prepared by dilution of a 0.1 M stock solution containing U(VI) and Th(IV) metal salts into the prepared nitric acid solutions to give a total aqueous phase volume of 2 mL. Toluene (2 mL) was added to each sample. Solid **L** (0.070 mmol) was added to a vial along with a magnetic stir bar (the order of addition of metal nitrate and **L** is not important). The mixture was stirred for 24 h at 298 K at 700 rpm, after which the stir bar was removed. Samples were prepared for ICP-MS to measure the metal content remaining in the aqueous phase (compared with the feed solution) to determine metal uptake by **L**. Samples were diluted in 2% nitric acid prior to ICP-MS analysis. These procedures were repeated in duplicate. Uncertainties associated with these results range between ±0.59 and ±9% precipitation.

#### 4.2.2 Precipitation Procedure for Np-237

Nitric acid solutions (0.8–8 M) were prepared by dilution of concentrated nitric acid with ultrapure deionized water. Toluene (2 mL) was added to each sample. Solid **L** (0.04 mmol) was added to the vial along with a magnetic stir bar. Np-237 solutions (474 ppm) were prepared by dilution of an ∼0.02 M stock solution of Np in nitric acid (8 M) into the prepared nitric acid solutions to give a total aqueous phase volume of 2 mL. To convert the small amounts of Np(VI) present in the original stock solution to Np(IV), 200 μL of H_2_O_2_ (30 wt %) was added, and the speciation was monitored through UV–vis spectroscopy (Supporting Information, Figure S13). The mixture was stirred for 24 h at 298 K at 700 rpm after which the stir bar was removed. Samples were prepared for α/α spectrometry to measure the metal content remaining in the aqueous phase (compared to the feed solution) to determine metal uptake by **L**. Samples were diluted in 2% nitric acid prior to analysis. These procedures were repeated in duplicate.

#### 4.2.3 Precipitation Procedure for Pu-238

Nitric acid solutions (0.8–8 M) were prepared by dilution of concentrated nitric acid with ultrapure deionized water. Toluene (2 mL) was added to each sample. Solid **L** (0.04 mmol) was added to the vial along with a magnetic stir bar. Pu-238 solutions (486 ppm) were prepared by dilution of an ∼0.008 M stock solution of Pu in nitric acid (1 M) into the prepared nitric acid solutions to give a total aqueous phase volume of 2 mL. To convert the small amounts of non-Pu(IV) present in the original stock solution to Pu(IV), 300 μL of H_2_O_2_ (30 wt %) was added, and the speciation was monitored through UV–vis spectroscopy, the stock solution was determined to be 90% Pu(IV) and 10% Pu(VI) (Supporting Information, Figure S14). The mixture was stirred for 24 h at 298 K at 700 rpm after which the stir bar was removed. Samples were prepared for α/α spectrometry and LSC to measure the metal content remaining in the aqueous phase (compared with the feed solution) to determine metal uptake by **L**. Samples were diluted in 2% nitric acid prior to analysis. These procedures were repeated in duplicate.

#### 4.2.4 Precipitation Procedure for Am-243 and Cm-245/248

Solid **L** (0.0095 mmol for Am, 0.01 mmol for Cm) was added to toluene (1 mL) along with a magnetic stir bar. To this, 1 mL of the metal stock solution (230 ppm for ^243^Am, 265 ppm total Cm) in nitric acid (8 M) was added. The nitric acid concentration of the metal stock solutions was adjusted through dilution with ultrapure deionized water to 6 M, and the amount of ligand added to these experiments was adjusted to maintain a 10-fold excess over the adjusted metal concentration. The mixtures were stirred for 24 h at 298 K at 700 rpm after which the stir bar was removed. Am samples were prepared for α/α spectrometry and Cm samples for both LSC and α/α spectrometry, to measure the metal content remaining in the aqueous phase (compared with the feed solution) to determine metal uptake by **L**. Samples were diluted in 2% nitric acid prior to analysis. These procedures were repeated in duplicate.

#### 4.2.5 Precipitation Procedure for Bk-249/Cf-249

Solid **L** (0.009 mmol) was added to toluene (0.5 mL) along with a magnetic stir bar. To this, 0.5 mL of the mixed Bk/Cf metal stock solution (373 ppm Bk, 244 ppm of Cf) in nitric acid (8 M) was added. The nitric acid concentration of the metal stock solution was adjusted through dilution with ultrapure deionized water to 6 M, and the amount of ligand added to these experiments was adjusted to maintain a 10-fold excess over the adjusted metal concentration. The mixtures were stirred for 24 h at 298 K at 700 rpm after which the stir bar was removed. Am samples were prepared for α/α spectrometry and LSC to measure the metal content remaining in the aqueous phase (compared with the feed solution) to determine metal uptake by **L**. Samples were diluted with 2% nitric acid prior to analysis. These procedures were repeated in duplicate.

#### 4.2.6 Precipitation Procedure for Fission/Corrosion Products

Nitric acid solutions (8 M) were prepared by dilution of concentrated nitric acid with ultrapure deionized water. Mixed-metal solutions (0.0025 M) were prepared by dilution of various Agilent ICP/AAS-standard solutions into prepared nitric acid solutions to give a total aqueous phase volume of 2 mL. Toluene (2 mL) was added to each sample. Solid **L** (0.070 mmol) was added to a vial along with a magnetic stir bar. The mixture was stirred for 24 h at 298 K at 700 rpm after which the stir bar was removed. Samples were prepared for ICP-MS to measure the metal content remaining in the aqueous phase (compared with the feed solution) to determine metal uptake by **L**. Samples were diluted in 2% nitric acid prior to ICP-MS analysis. These procedures were repeated in duplicate.

#### 4.2.7 Stripping Experiments

Nitric acid solutions (8 M) were prepared by dilution of concentrated nitric acid with ultrapure deionized water. Th(NO_3_)_4_ (0.025 M) solutions were prepared by dilution of a 0.1 M stock solution containing thorium nitrate into the prepared nitric acid solutions to give a total aqueous phase volume of 2 mL. Toluene (2 mL) was added to each sample. Solid **L** (0.05 mol) was added to a vial along with a magnetic stir bar. The mixture was stirred for 24 h at 298 K at 700 rpm after which the stir bar was removed. Samples were prepared for ICP-MS to measure the metal content remaining in the aqueous phase (compared with the feed solution) to determine metal uptake by **L**. Samples were diluted by 2000× in 2% nitric acid prior to ICP-MS analysis. The metal-containing precipitate was collected through filtration and washed with water (4 mL) for 1 h. The solid ligand was collected by filtration and the strip solution was analyzed as before.

## Data Availability

X-ray data are available free of charge from the Cambridge Crystallographic Data Centre (https://www.ccdc.cam.ac.uk/data_request/cif) under reference numbers CCDC 2294754–2294759. The quantitative metal analyses, IR, Raman, and NMR data are available upon request. The authors declare that all other data supporting the findings of this study are available within the paper and its Supporting Information files.

## Supporting Information Available

SCXRD tables, additional crystal structures, QTAIM analysis, change density difference plots, IR and Raman characterization, UV–vis/NIR spectra, and further metal precipitation data (PDF)
