# Supplementary material for: Shape-Selective Supramolecular Capsules for Actinide Precipitation and Separation
Source: JACS Au. 2024 Feb 12;4(2):798–806. doi: 10.1021/jacsau.3c00793 (PMC10900489; doi:10.1021/jacsau.3c00793)
Supplement: Supplementary file 1 — au3c00793_si_001.pdf [file au3c00793_si_001.pdf]

Supplementary Information: Shape-selective supramolecular capsules for actinide precipitation and separation

Joseph O'Connell-Danes,[1] Bryne T. Ngwenya,[2] Carole A. Morrison,[1] Gary S. Nichol,[1] Lætitia H. Delmau,[3]* and Jason B. Love[1]*

[1] EaStCHEM School of Chemistry, University of Edinburgh, Edinburgh, EH9 3FJ, U.K.

[2] School of Geosciences, University of Edinburgh, Edinburgh, EH9 3FE, U.K.

[3] Radioisotope Science and Technology Division, Oak Ridge National Laboratory, Oak Ridge, Tennessee 37831, USA.

*Corresponding authors: jason.love@ed.ac.uk; delmaulh@ornl.gov

1. Crystallographic Data

Supplementary Information Table 1. Crystallographic data for 1-Pu^{239}.

	**1-Pu^{239}**
Crystal data	
Chemical formula	$C_{84}H_{152}N_{12}O_{26}Pu$
M_r	1988.17
Crystal system, space group	Trigonal, $R\bar{3}$
Temperature (K)	302
a, *c* (Å)	14.0895(2), 44.7412(13)
V ($Å^3$)	7691.8(3)
Z	3
Radiation type	Mo *Kα*
μ (mm^{-1})	0.72
Crystal size (mm)	Not possible due to crystal visibility in epoxy and sheath required for mounting.
Data collection	
Diffractometer	Bruker *APEX*-II CCD
Absorption correction	Multi-scan<br>*SADABS2016*/2 (Bruker,2016/2) was used for absorption correction. wR2(int) was 0.1717 before and 0.0555 after correction. The Ratio of minimum to maximum transmission is 0.7861.
T_{min}, T_{max}	0.586, 0.746
No. of measured, independent and observed [I > 2□(I)] reflections	19483, 3646, 3599
R_{int}	0.043
$(\sin q/l)_{max}$ ($Å^{-1}$)	0.633
Refinement	
$R[F^2 > 2s(F^2)]$, $wR(F^2)$, S	0.028, 0.072, 1.07
No. of reflections	3646
No. of parameters	260
No. of restraints	204
H-atom treatment	H atoms treated by a mixture of independent and constrained refinement
$D\rho_{max}$, $D\rho_{min}$ (e $Å^{-3}$)	0.66, -0.24

Supplementary Information Table 2. Crystallographic data for 1-Np.

	**1-Np**
Crystal data	
Chemical formula	$C_{84}H_{152}N_{12}O_{26}Np$
M_r	1983.17
Crystal system, space group	Trigonal, $R\bar{3}$
Temperature (K)	301
a, *c* (Å)	14.0720(7), 44.673(3)
V (Å^3)	7661.0(9)
Z	3
Radiation type	Mo *Kα*
μ (mm^{-1})	1.09
Crystal size (mm)	Not possible – see Pu-239 details
Data collection	
Diffractometer	Bruker *APEX*-II CCD
Absorption correction	Multi-scan<br>*SADABS2016*/2 (Bruker,2016/2) was used for absorption correction.
No. of measured, independent and observed [*I* > 2□(*I*)] reflections	34582, 5203, 5039
R_{int}	0.055
(sin q/l)$_{max}$ (Å^{-1})	0.714
Refinement	
$R[F^2 > 2s(F^2)]$, $wR(F^2)$, S	0.031, 0.079, 1.07
No. of reflections	5203
No. of parameters	260
No. of restraints	545
H-atom treatment	H atoms treated by a mixture of independent and constrained refinement

Supplementary Information Table 3. Crystallographic data for 1-Th.

	**1-Th**
Crystal data	
Chemical formula	$C_{84}H_{152}N_{12}O_{26}Th$
M_r	1982.24
Crystal system, space group	Trigonal, $R\bar{3}$
Temperature (K)	120
a, *c* (Å)	14.0483(4), 44.0643(12)
V ($Å^3$)	7531.2(5)
Z	3
Radiation type	Mo *Kα*
μ (mm^{-1})	1.56
Crystal size (mm)	0.29 × 0.20 × 0.14
Data collection	
Diffractometer	SuperNova, Dual, Cu at home/near, Atlas
Absorption correction	Multi-scan<br>*CrysAlis PRO* 1.171.41.123a (Rigaku Oxford Diffraction, 2022) Empirical absorption correction using spherical harmonics, implemented in SCALE3 ABSPACK scaling algorithm.
T_{min}, T_{max}	0.917, 1.000
No. of measured, independent and observed [*I* > 2□(*I*)] reflections	22863, 5764, 5275
R_{int}	0.041
$(\sin q/l)_{max}$ ($Å^{-1}$)	0.765
Refinement	
$R[F^2 > 2s(F^2)]$, $wR(F^2)$, *S*	0.048, 0.128, 1.07
No. of reflections	5764
No. of parameters	259
No. of restraints	99
H-atom treatment	H atoms treated by a mixture of independent and constrained refinement

Supplementary Information Table 4. Crystallographic data for 1-Ce.

	**1-Ce**
Crystal data	
Chemical formula	$CeN_6O_{18}·C_{84}H_{153}N_6O_6·1[C2H3N]$
M_r	1855.29
Crystal system, space group	Trigonal, $R\bar{3}$
Temperature (K)	100
a, *c* (Å)	21.2755(4), 20.3388(4)
V ($Å^3$)	7972.9(3)
Z	3
Radiation type	Cu *Kα*
m (mm^{-1})	3.87
Crystal size (mm)	0.24 × 0.19 × 0.10 × 0.10 (radius)
Data collection	
Diffractometer	SuperNova, Dual, Cu at home/near, Atlas
Absorption correction	For a sphere<br>*CrysAlis PRO* 1.171.41.123a (Rigaku Oxford Diffraction, 2022) Spherical absorption correction using equivalent radius and absorption coefficient. Empirical absorption correction using spherical harmonics, implemented in SCALE3 ABSPACK scaling algorithm.
T_{min}, T_{max}	0.578, 0.610
No. of measured, independent and observed [I > 2s(I)] reflections	14215, 3634, 3629
R_{int}	0.051
(sin q/l)$_{max}$ ($Å^{-1}$)	0.629
Refinement	
$R[F^2 > 2s(F^2)]$, $wR(F^2)$, S	0.044, 0.117, 1.07
No. of reflections	3634
No. of parameters	262
No. of restraints	82
H-atom treatment	H atoms treated by a mixture of independent and constrained refinement
Dρ$_{max}$, Dρ$_{min}$ (e $Å^{-3}$)	0.77, -1.13

Supplementary Information Table 5. Crystallographic data for 1-Am.

	**1-Am**
Crystal data	
Chemical formula	$AmN_6O_{18}\cdot C_{84}H_{153}N_6O_6$
M_r	1958.17
Crystal system, space group	Trigonal, $R\bar{3}$
Temperature (K)	299
a, *c* (Å)	21.4573(11), 20.6563(19)
V (Å^3)	8236.3(11)
Z	3
Radiation type	Mo *Kα*
m (mm^{-1})	0.76
Crystal size (mm)	Not available, see previous comments.
Data collection	
Diffractometer	Bruker *SMART APEX2* area detector
Absorption correction	Multi-scan *SADABS2016*/2 (Bruker,2016/2) was used for absorption correction. wR2(int) was 0.1212 before and 0.1013 after correction. The Ratio of minimum to maximum transmission is 0.6210. The l/2 correction factor is Not present.
T_{min}, T_{max}	0.462, 0.744
No. of measured, independent and observed [$I > 2s(I)$] reflections	9207, 1115, 1071
R_{int}	0.108
q_{max} (°)	17.2
$(\sin q/l)_{max}$ (Å^{-1})	0.416
Refinement	
$R[F^2 > 2s(F^2)]$, $wR(F^2)$, S	0.035, 0.082, 1.09
No. of reflections	1115
No. of parameters	242
No. of restraints	211
H-atom treatment	H atoms treated by a mixture of independent and constrained refinement
$D\rho_{max}$, $D\rho_{min}$ (e Å^{-3})	0.41, -0.31

Supplementary Information Table 6. Crystallographic data for 1-Eu.

	**1-Eu**
Crystal data	
Chemical formula	$EuN_6O_{18}·C_{84}H_{153}N_6O_6$
M_r	1867.13
Crystal system, space group	Trigonal, $R\bar{3}$
Temperature (K)	100
a, *c* (Å)	21.3081(4), 20.2158(5)
V (Å^3)	7949.0(4)
Z	3
Radiation type	Cu $K\alpha$
m (mm^{-1})	4.79
Crystal size (mm)	0.23 × 0.20 × 0.06 × 0.08 (radius)
Data collection	
Diffractometer	SuperNova, Dual, Cu at home/near, Atlas
Absorption correction	For a sphere<br>*CrysAlis PRO* 1.171.41.123a (Rigaku Oxford Diffraction, 2022) Spherical absorption correction using equivalent radius and absorption coefficient. Empirical absorption correction using spherical harmonics, implemented in SCALE3 ABSPACK scaling algorithm.
T_{min}, T_{max}	0.477, 0.520
No. of measured, independent and observed [I > 2s(I)] reflections	14336, 3655, 3583
R_{int}	0.068
(sin q/l)$_{max}$ (Å^{-1})	0.629
Refinement	
$R[F^2 > 2s(F^2)]$, $wR(F^2)$, S	0.053, 0.132, 1.09
No. of reflections	3655
No. of parameters	254
No. of restraints	14
H-atom treatment	H atoms treated by a mixture of independent and constrained refinement
Dρ_{max}, Dρ_{min} (e Å^{-3})	1.17, -0.88

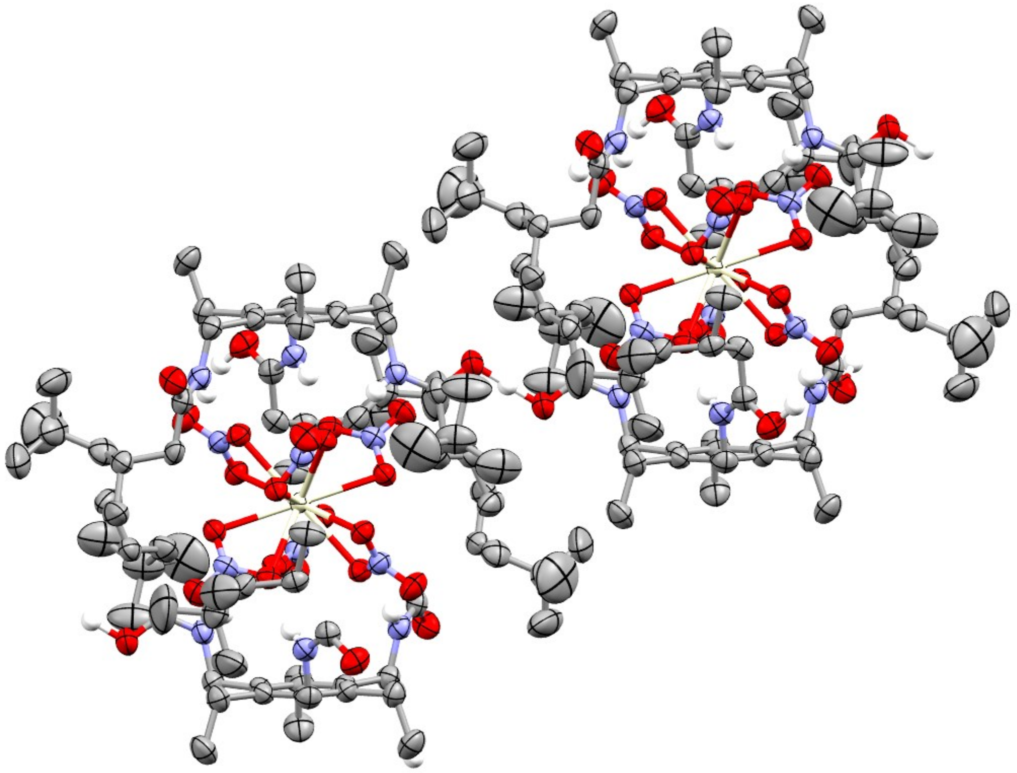

Supplementary Figure 1. X-ray crystal structure of 1-Ce (side-on view). For clarity, all hydrogen atoms except those involved in hydrogen bonding and a disorder component of the amide arm are omitted (thermal displacement ellipsoids are drawn at 50% probability). Atom colours: Ce = cream; oxygen = red; nitrogen = blue; carbon = silver; hydrogen = white.

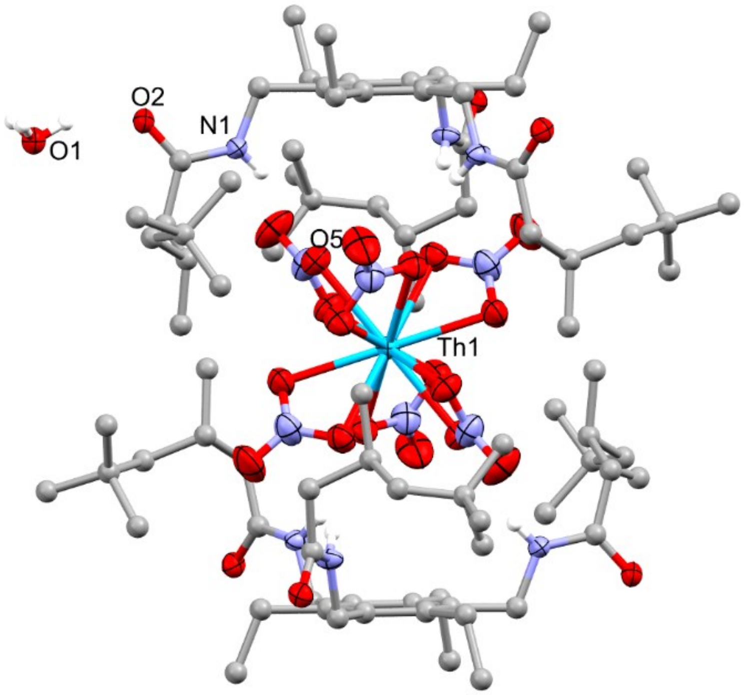

Supplementary Figure 2. X-ray crystal structure of 1-Th (side-on view). For clarity, all hydrogen atoms except those involved in hydrogen bonding and a disorder component of the amide arm are omitted (thermal displacement ellipsoids are drawn at 50% probability). N-H and O-H hydrogen atoms were located in the difference Fourier map and (O1) is 33.33% occupied on a crystallographic special position. Atom colours: Th = blue; oxygen = red; nitrogen = blue; carbon = silver; hydrogen = white.

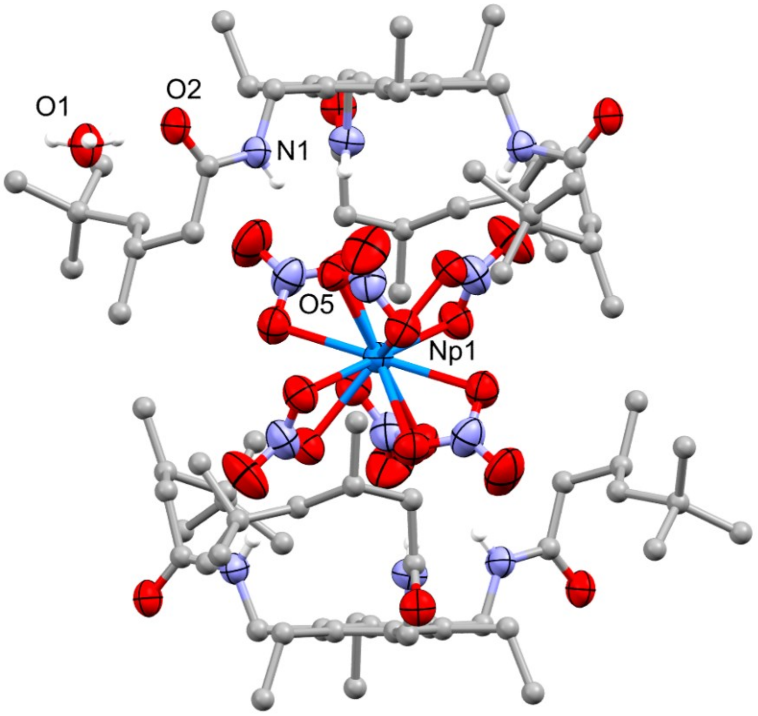

Supplementary Figure 3. X-ray crystal structure of 1-Np (side-on view). For clarity, all hydrogen atoms except those involved in hydrogen bonding and a disorder component of the amide arm are omitted (thermal displacement ellipsoids are drawn at 50% probability). N-H and O-H hydrogen atoms were located in the difference Fourier map and (O1) is 33.33% occupied on a crystallographic special position. Atom colours: Np = blue; oxygen = red; nitrogen = blue; carbon = silver; hydrogen = white.

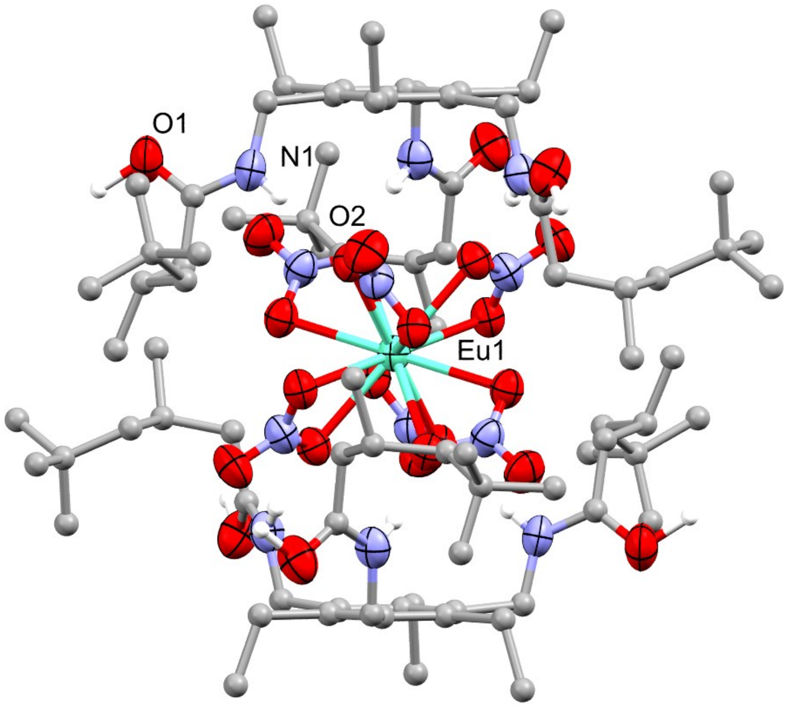

Supplementary Figure 4. X-ray crystal structure of 1-Eu (side-on view). For clarity, all hydrogen atoms except those involved in hydrogen bonding and a disorder component of the amide arm are omitted (thermal displacement ellipsoids are drawn at 50% probability). Atom colours: Eu = teal; oxygen = red; nitrogen = blue; carbon = silver; hydrogen = white.

2. Computational analysis

Supplementary Information Table 7. Key results from QTAIM analysis of the DFT optimised structures for 1-Ce, 1-Th, 1-Np, and 1-Pu.

**Metal**	**Ce**	**Th**	**Np**	**Pu**
**Bond lengths/Å**				
*r*M-ONO_2	2.642/2.648	2.560/2.596	2.509/2.550	2.484/2.529
Intra-capsule H-bond *r*N-H…O	2.816	3.247	3.333	3.373
External-capsule H-bond *r*O…H…O	2.424	–	–	–
External-capsule H-bond *r*O…HOH_2	–	2.545	2.547	2.555
**QTAIM ρ_{bcp} / eÅ^{-3}**				
*r*M…ONO_2	0.243/0.243	0.353/0.325	0.362/0.327	0.371/0.332
Intra-capsule H-bond *r*N-H…O	0.252	0.094	0.077	0.069
External-capsule H-bond *r*O..H…O	1.189	–	–	–
External-capsule H-bond *r*O…HOH_2	–	0.541	0.536	0.526

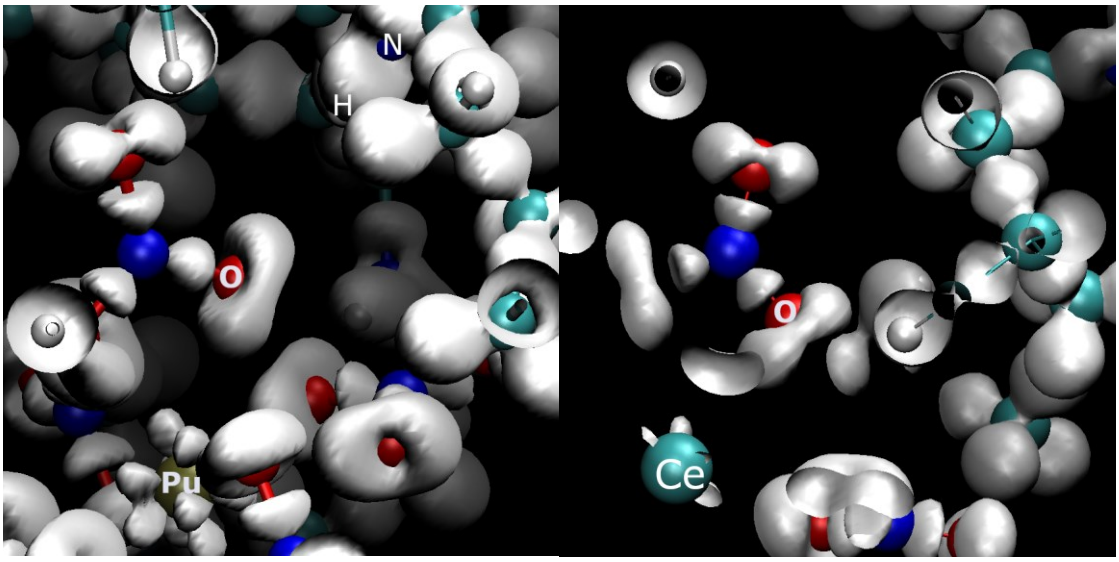

Supplementary Figure 5. Charge density difference plots of 1-Pu (left) and 1-Ce (right), ISO value set at 0.000125. Plots show the difference between the calculated electron density and the atomic densities and thus highlight areas of bonding electrons and lone pairs.

3. Spectroscopic characterisation

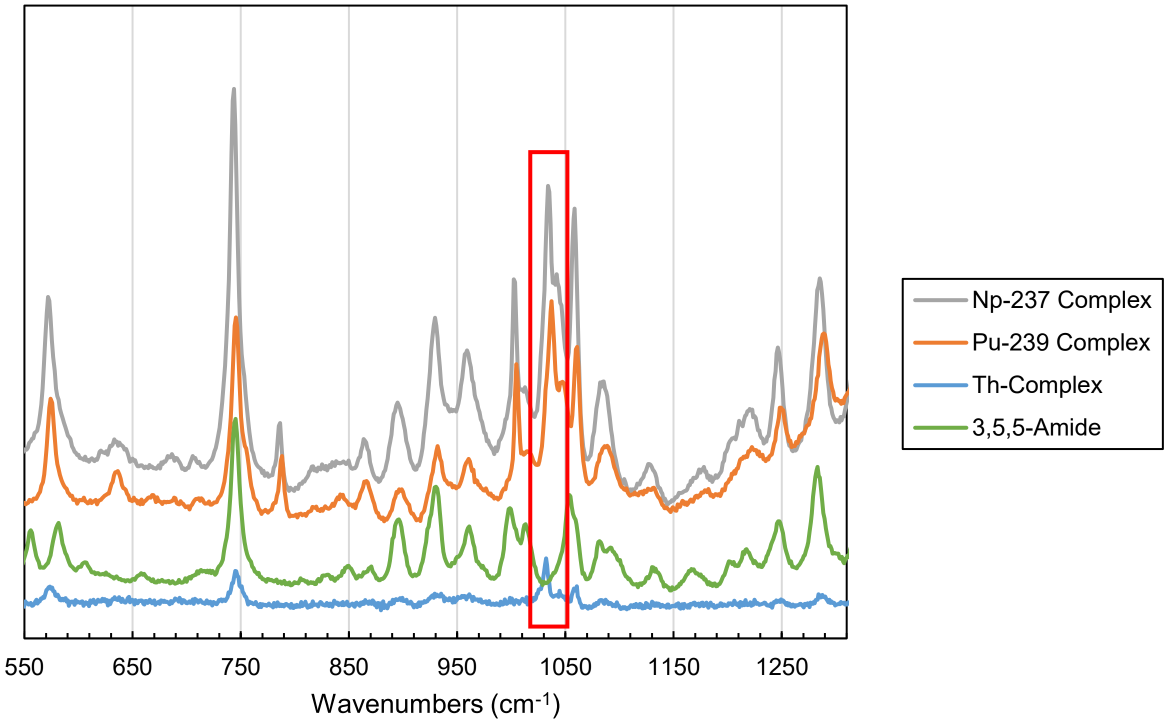

Supplementary Figure 6. Comparison of the Raman spectra of the free ligand L (Green), with Th (blue), Pu (orange), and Np (grey) containing precipitates. The red box highlights the symmetric stretching vibration (ν_s) of the metal-bound NO_3^- groups in the hexanitratometalates.

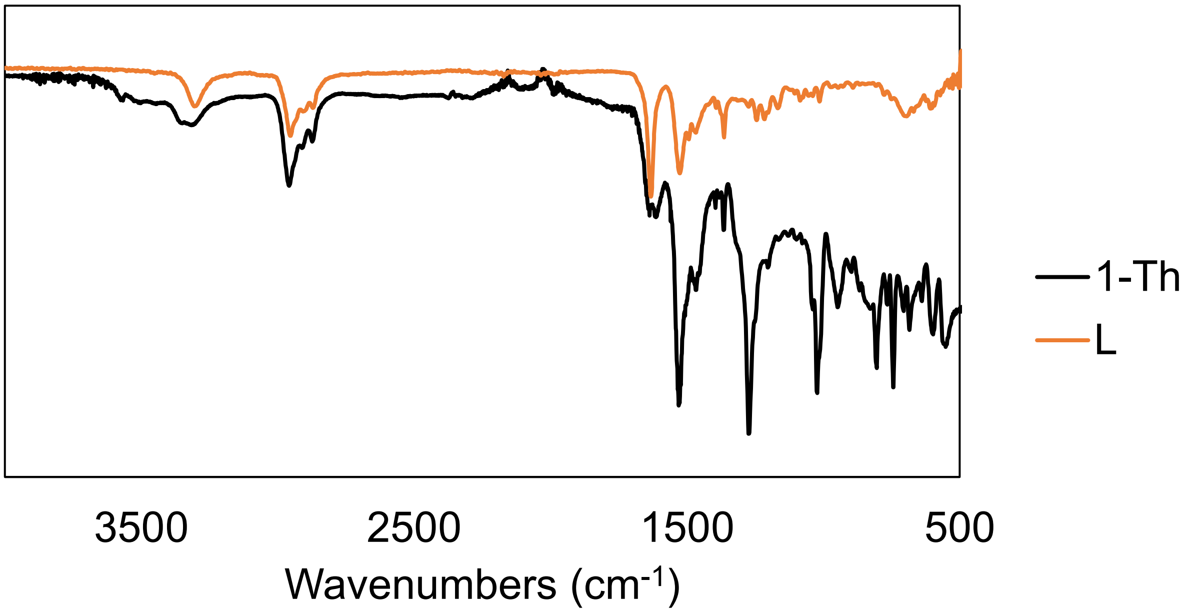

Supplementary Figure 7. Infrared spectra of 1-Th (black) and L (orange).

4. UV-VIS/NIR Spectra

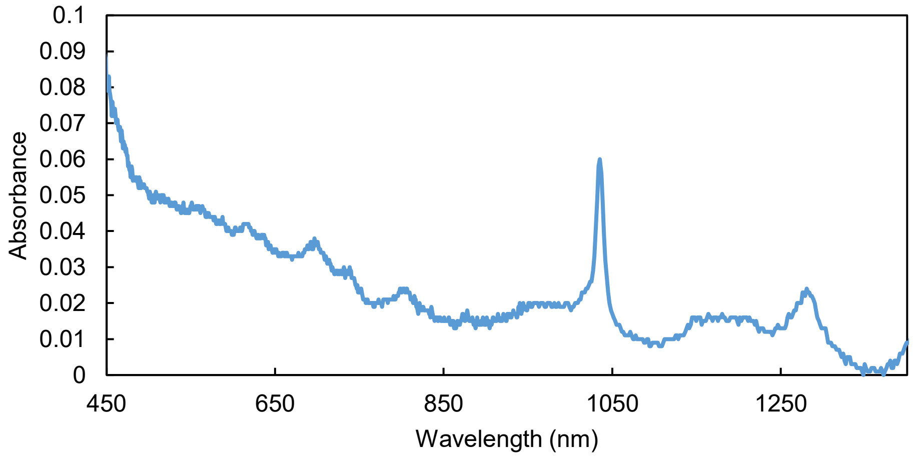

Supplementary Figure 8. UV-VIS/NIR of Np(V/VI) solution in 8 M HNO_3.

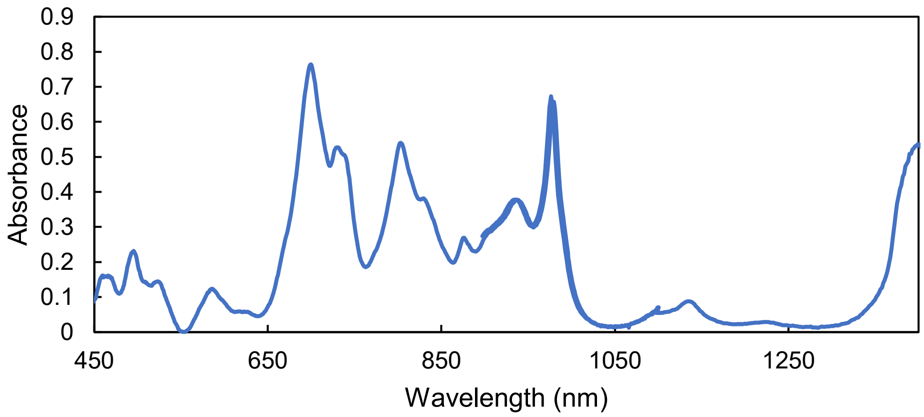

Supplementary Figure 9. UV-VIS/NIR of Np(IV) solution in 8 M HNO_3.

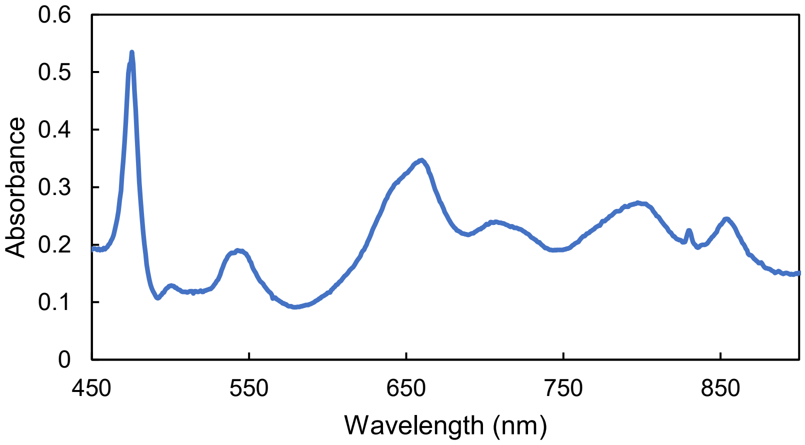

Supplementary Figure 10. UV-VIS of Pu(IV) solution in 8 M HNO_3.

5. Metal precipitation data

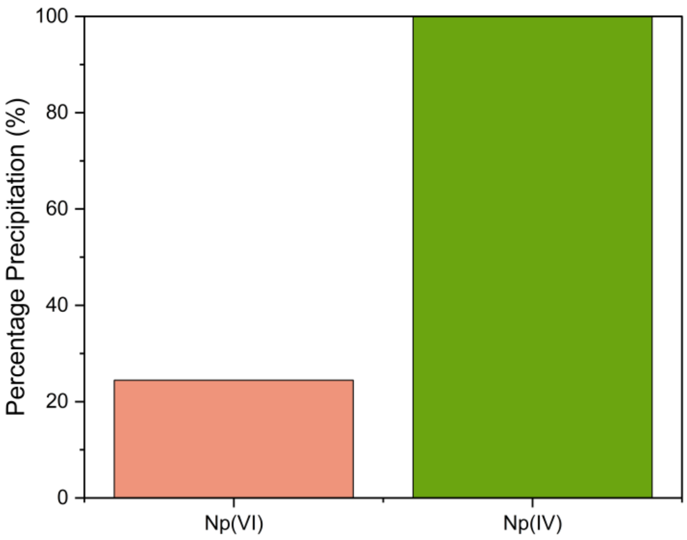

Supplementary Figure 11. Precipitation arising from single metal solutions of ^{237}Np (474 ppm) in 8 M HNO_3/toluene equal-volume biphasic mixture after the addition of L (10-fold excess L relative to metal) at 298 K. The Np(VI) was determined to be made up of 71% Np(VI), 10% Np(V), and 19% Np(IV). The Np(IV) solution was determined to contain only Np(IV) after the addition of hydrogen peroxide.

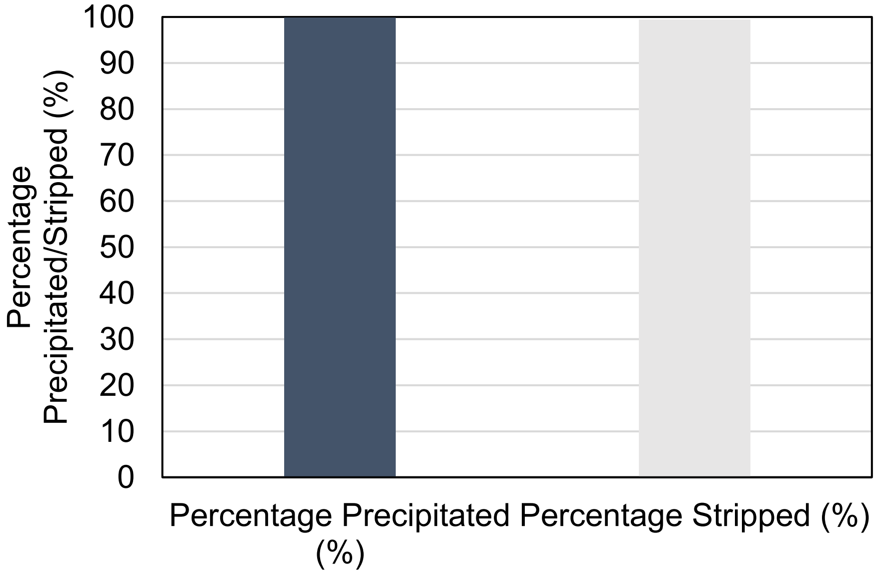

Supplementary Figure 12. Precipitation of thorium nitrate by L from 8 M HNO3 (Precipitated) follow by its release using water (Stripped).

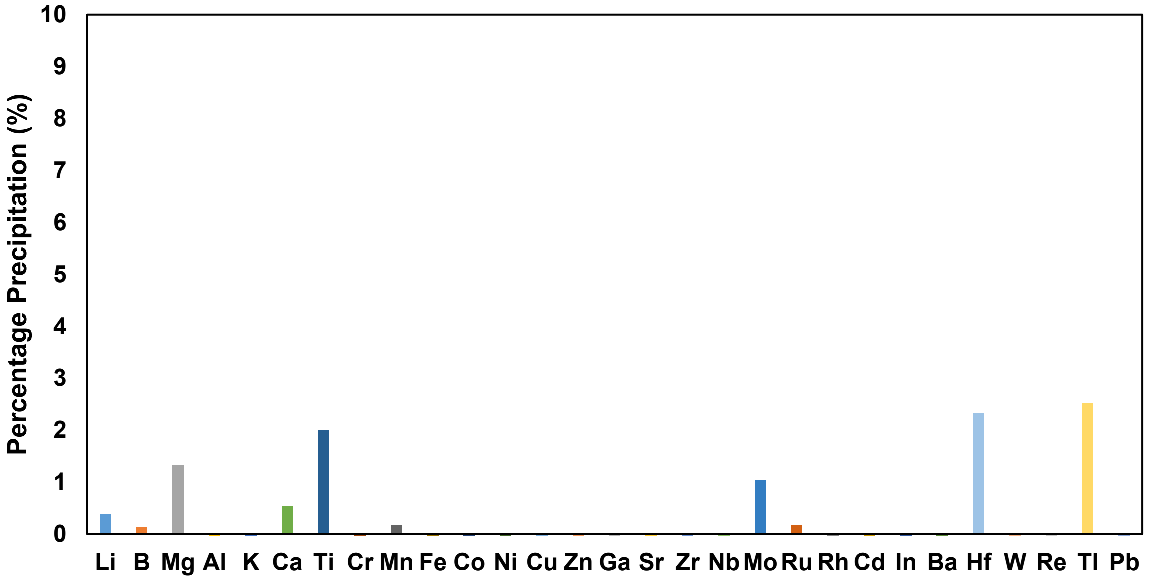

Supplementary Figure 13. Precipitation arising from mixed-metal solutions (500 ppm) in 8.0 M HNO_3/toluene equal-volume biphasic mixture after the addition of L (5-fold excess L relative to metal) at 298 K.
